# Cancer Prevention in Adults with Intellectual Disabilities: A Systematic Literature Review of Caregiver Perspectives in Institutional and Home Care Settings

**DOI:** 10.3390/ijerph21111402

**Published:** 2024-10-23

**Authors:** Simon Klara, Mohammed Elmadani, Horváth Éva, Tóth Lívia, Godfrey Mbaabu, Osama F. Hamad, Amer Mesmar, Diego Andrade, Orsolya Mate

**Affiliations:** 1 Doctoral School of Health Sciences, Faculty of Health Sciences, University of Pecs, 7622 Pecs, Hungary; klara.simon@etk.pte.hu (S.K.); eva.horvath@etk.pte.hu (H.É.); livia.toth@etk.pte.hu (T.L.); mbaabugodfrey3@gmail.com (G.M.); ussama_90@hotmail.com (O.F.H.); amerrmismarr@gmail.com (A.M.); 2 Faculty of Health Sciences, University of Pecs, 7622 Pecs, Hungary; andrade.diego@etk.pte.hu

**Keywords:** intellectual disability, cancer screening, caregiver, healthcare disparities, intervention

## Abstract

**Background:** Individuals with intellectual disabilities (IDs) face unique challenges in accessing cancer prevention measures. Caregivers play a crucial role in facilitating these measures, yet their perspectives are under-researched. **Methods:** This systematic literature review explores caregiver perspectives on cancer prevention for adults with IDs in institutional and home care settings, aiming to understand their roles, knowledge, and challenges. Thirteen articles from the UK, the USA, Canada, and Ireland were analyzed through thematic synthesis. Four overarching themes were identified: breast cancer screening perspectives, caregivers’ knowledge and barriers to cancer prevention, caregivers’ perspectives, and cultural context and decision-making. **Results:** Caregivers, including healthcare professionals and family members, are essential in facilitating breast cancer screening for individuals with IDs. Challenges include explaining screening procedures, limited awareness, and logistical barriers, highlighting the need for targeted educational interventions. Disparities in access underscore the necessity for comprehensive training programs. Healthcare professionals’ perspectives reveal existing disparities and suggest interventions for improved accessibility and understanding. Cultural context influences decision-making, emphasizing the importance of culturally sensitive care. The role of family caregivers in decision-making necessitates empowering and supporting them through tailored interventions. **Conclusions:** This review provides insights into the challenges and opportunities in cancer prevention for individuals with IDs, suggesting the need for educational interventions, training programs, and systemic changes to address disparities. It lays the groundwork for future research and the development of holistic and inclusive strategies in this critical healthcare domain.

## 1. Introduction

Cancer prevention stands as a crucial pillar of healthcare, addressing diverse populations with unique challenges and considerations [1]. Among these, adults with intellectual disabilities (IDs) emerge as a distinct group requiring special attention due to the multifaceted nature of their needs [2,3,4]. This literature review delves into caregiver perspectives on cancer prevention in this demographic, examining the roles and knowledge of caregivers in both institutional and home care settings. Recognizing the intersection of intellectual disabilities and cancer prevention is pivotal for tailoring inclusive healthcare strategies that effectively address the specific challenges faced by this vulnerable group [3].

Intellectual disabilities encompass a spectrum of cognitive impairments impacting adaptive functioning, communication, and daily living skills [5]. The heightened vulnerability within this demographic arises not only from cognitive challenges but also from a complex interplay of limited access to healthcare resources, communication barriers, and societal misconceptions [6,7,8,9]. Caregivers play a central role amid these challenges, navigating the intricate web of care responsibilities and knowledge dissemination [6,7]. The distinction between institutional and home care settings introduces further complexity to the caregiving landscape [10]. Institutional caregivers provide structured environments in group homes or residential facilities, while home caregivers manage diverse responsibilities, striving to create inclusive and comfortable spaces [11].

Understanding how caregivers engage in cancer prevention within these settings is essential for tailoring interventions to the unique needs of adults with intellectual disabilities [12,13,14,15,16]. This literature review synthesizes existing knowledge on caregiver perspectives regarding cancer prevention within the context of intellectual disabilities, aiming to provide insights that inform policy, practice, and future research. Enhancing our understanding of caregiver perspectives is crucial for developing inclusive and effective healthcare strategies tailored to this underserved population [1,17].

The justification for selecting adults with intellectual disabilities for cancer prevention research lies in their unique health disparities, particularly in cancer screening and prevention. Caregivers in both institutional and home settings play a critical role in identifying early symptoms and promoting cancer prevention. However, they often lack the necessary training to support these efforts effectively. A significant gap exists in age-specific cancer screening for adults with developmental disabilities, where caregivers are essential for ensuring adherence to preventive measures. Training for caregivers is recommended to improve access to screenings and other health services [18]. Additionally, unified cancer prevention policies tailored to the specific needs of individuals with IDs can enhance screening rates and health behaviors [1].

Despite the challenges presented by intellectual disabilities, including cognitive impairments affecting adaptive functioning, communication, and daily living skills [9,19,20], the lack of cancer-related knowledge among care staff further complicates prevention efforts. Training caregivers on cancer risk factors and health promotion is vital, as cognitive impairments in individuals with IDs make early detection challenging [21]. Caregiver perspectives are essential; adults with IDs rely on them for healthcare needs, including access to screenings and appointments. Without informed caregivers, adults with IDs may experience delays in cancer detection and lower participation in preventive behaviors [18]. Caregivers also help interpret health information, recognize early warning signs, and advocate for screenings, making their insights critical for creating accessible health promotion materials [21]. Many caregivers report inadequate training regarding cancer prevention for people with IDs. Including their perspectives can identify specific training needs, facilitating targeted educational programs that empower caregivers to promote health and early detection. Improving caregiver knowledge about cancer risk factors can significantly impact outcomes for individuals with IDs [1].

Furthermore, caregiver insights shape effective health policies and practices. Policies that incorporate caregivers’ experiences can better address the challenges faced in caregiving. Involving caregivers in policy discussions ensures that strategies for cancer prevention are practical and applicable [1]. Adults with IDs often receive late-stage diagnoses for cancers like breast and colorectal cancer, complicating treatment and reducing survival rates [22]. Limited data on the prevalence and incidence of cancer in individuals with IDs underscores the need for systematic reviews to understand the burden of cancer in this population [22]. Some studies indicate that certain cancers, particularly digestive tract cancers, occur earlier in individuals with profound disabilities compared to the general population, necessitating tailored, age-appropriate prevention measures [23].

The prevalence of cancer among individuals with IDs is complex; studies show lower overall cancer rates but increased risks for specific types, such as brain tumors and leukemia [24]. Barriers to healthcare access lead to later-stage diagnoses and poorer outcomes [25]. Adults with IDs often receive less intensive treatments than typically recommended, contributing to higher mortality rates and underscoring the need for tailored treatment plans that address their unique challenges [26].

These issues contribute to heightened vulnerability, exposing individuals to an array of health disparities shaped by limited access to healthcare resources, communication barriers, and societal misconceptions [9,27]. Understanding the distinctive health challenges faced by this population is vital, particularly given the increased prevalence of comorbid conditions [1]. Adults with intellectual disabilities often bear a higher burden of chronic health issues, predisposing them to elevated risks of various types of cancer. This necessitates a nuanced approach to cancer prevention tailored to their unique healthcare needs [21].

The umbrella of intellectual disabilities encompasses conditions like Down syndrome and intellectual developmental disorders, each requiring tailored healthcare strategies for both cognitive aspects and associated health challenges [28]. This vulnerability is further compounded by a complex interplay of socioeconomic factors, discrimination, and barriers within the healthcare system [8]. The lack of tailored healthcare services and the scarcity of providers with expertise in intellectual disabilities contribute to delayed diagnoses and the inadequate management of health conditions [29]. Against this backdrop, cancer prevention emerges as a critical component of healthcare for adults with intellectual disabilities, demanding a proactive and targeted approach [30].

In navigating this complex healthcare landscape, caregivers play an indispensable role. Beyond providing daily support, caregivers become advocates for health equity, actively participating in cancer prevention efforts. Their responsibilities include promoting awareness, facilitating regular screenings, and implementing preventive measures tailored to the specific needs of individuals with intellectual disabilities [4,31,32,33].

Current cancer prevention policies do not adequately meet the unique needs of individuals with IDs, resulting in lower participation in routine screenings. Factors such as communication barriers and transportation issues exacerbate these disparities. Therefore, targeted cancer prevention strategies for this population are essential [1]. Closing this research gap requires robust data collection, tailored cancer prevention strategies, enhanced caregiver training, inclusive health policies, and increased awareness among healthcare providers regarding the disparities faced by this vulnerable population.

This study aims to elucidate caregiver perspectives on cancer prevention in adults with intellectual disabilities in institutional and home care settings. This aim will be achieved through the following objectives:

- Explore and analyze the specific roles that caregivers play in the context of cancer prevention for adults with intellectual disabilities and assess their knowledge regarding tailored prevention strategies.
- Identify and analyze the specific challenges faced by caregivers in the effective implementation of cancer prevention measures for adults with intellectual disabilities. Additionally, this study scrutinizes existing literature gaps related to the role and knowledge of caregivers in cancer prevention for intellectually disabled adults, proposing areas that require further research or intervention for enhanced understanding and support in this domain.

## 2. Materials and Methods

A comprehensive literature search was conducted from October 2023 to January 2024 to identify relevant studies, including published papers and conference abstracts. The search covered multiple databases, such as MEDLINE (Ovid), EMBASE, PubMed, CINAHL, and Web of Science. Both electronic and manual methods were utilized to identify reference lists of included papers, along with a snowball search technique to uncover additional relevant studies based on the references of initially identified articles.

The authors of selected studies focusing on caregivers’ perspectives were contacted via email or other available communication methods to solicit additional information if needed. The search was confined to English-language databases to maintain consistency.

Keywords and their synonyms were utilized, including “Cancer prevention,” “Intellectual disabilities”, “Caregivers”, “Institutional care”, and “Home care” (Appendix A). Specific inclusion criteria were established to ensure relevance, which included the following: (1) studies involving caregivers of individuals with intellectual disabilities; (2) studies focusing on cancer prevention in adults with intellectual disabilities; (3) studies that include perspectives of caregivers on cancer prevention; (4) studies reported among institutional or home care settings; and (5) only English-language publications were considered due to practical constraints. Exclusion criteria filtered out studies that did not focus on cancer prevention or caregiver perspectives.

This review encompassed a diverse array of study designs to explore caregivers’ roles and knowledge in cancer prevention for adults with intellectual disabilities. Quantitative studies, including randomized controlled trials, cohort studies, and case–control studies, assessed intervention effectiveness and explored associations between caregiver involvement and cancer prevention outcomes. Qualitative studies, such as phenomenological and grounded theory studies, provided insights into caregivers’ lived experiences. Mixed-method studies, intervention studies, cross-sectional studies, and descriptive studies were also included to capture both quantitative and qualitative aspects of caregiver engagement. Systematic reviews and meta-analyses were excluded from this review.

Two independent reviewers systematically conducted blinded title and abstract screening, full-text screening, and data extraction using Cochrane Review Manager (Covidence). To manage potential biases during the selection process, discrepancies were resolved through discussion, ensuring that included studies met the predetermined criteria. A standardized form was employed to collect pertinent information from selected studies, including study information (authors, publication year, title), study design, and key findings (Table 1). Data extraction was performed independently by two reviewers, with discrepancies resolved through discussion or consultation with a third reviewer if needed (Appendix B).

A PRISMA (Preferred Reporting Items for Systematic Reviews and Meta-Analyses) flowchart was used to visually represent the selection process for the included studies. This flowchart illustrates the number of records identified, screened, excluded, and ultimately included in the review. Its inclusion provides transparency in the study selection process and highlights how the final set of studies was determined. By detailing each step of the process, such as the reasons for exclusions at various stages, the PRISMA flowchart plays a crucial role in ensuring a clear and systematic interpretation of the results.

The quality assessment of the included studies was conducted using the Mixed Methods Appraisal Tool (MMAT) [34]. This tool is designed to evaluate the methodological quality of studies with diverse designs, encompassing qualitative, quantitative, and mixed-method studies. A rating of 4 stars or more is indicative of high quality; 3 stars denote moderate quality, while studies receiving fewer than 3 stars are considered low quality (Table 1). Data synthesis was conducted systematically, integrating findings from diverse study designs, including quantitative, qualitative, and mixed-method studies. A narrative and thematic synthesis approach was employed to present and summarize key themes, patterns, and variations across studies. The thematic synthesis was conducted using Thomas and Harden’s (2008) [35] approach to analyze caregiver perspectives on cancer prevention in adults with intellectual disabilities in both institutional and home care settings. This method facilitated the systematic identification of key themes relevant to the study’s objectives, focusing on caregivers’ roles, knowledge, and challenges. The first step involved the free coding of qualitative data from the selected studies, where the research team performed line-by-line coding to capture concepts related to caregivers’ involvement in cancer prevention, their knowledge of tailored strategies, and their practical experiences. Example codes included “caregivers’ involvement in health screening”, “lack of knowledge about cancer risks”, and “difficulty accessing tailored prevention information”. After coding, related concepts were grouped into descriptive themes addressing caregivers’ roles, their knowledge about prevention strategies, and barriers to implementation. For instance, codes related to limited knowledge among caregivers and healthcare professionals regarding cancer prevention and those concerning practical challenges in accessing and navigating the screening process were grouped under the theme “caregivers’ knowledge and barriers to cancer prevention”. Similarly, codes addressing the role of healthcare professionals in mitigating disparities led to the theme “caregivers’ perspectives,” and those highlighting the influence of cultural context on decision-making were grouped under the theme “cultural context and decision-making”. In the final stage, the descriptive themes were synthesized into broader analytical themes that provided deeper insights into the systemic and practical challenges caregivers face, including the need for institutional interventions. Additionally, the synthesis highlighted gaps in the literature requiring further research or targeted support. To ensure credibility and validity, the coding and themes were independently reviewed by multiple researchers, with discrepancies resolved through discussion, ensuring that the final themes accurately reflected the data and aligned with the study’s objectives.

In the preparation of this manuscript, ChatGPT, an AI-assisted tool, was used to support language editing and paraphrasing. After utilizing the tool, all content was manually reviewed to ensure clarity, accuracy, and alignment with the intended meaning. The AI tool did not contribute to the intellectual content or conceptualization of the work. The study protocol has been registered with Open Science Framework (https://osf.io/, accessed on 23 June 2024) with the documentation number [DOI: https://doi.org/10.17605/OSF.IO/5F6NG] (accessed on 23 June 2024).

**Table 1:** Data extracted from reported articles (*n* = 13).

Study, Country	Aim	Type of Study	Key Findings	Conclusion	Quality
[36] Scotland	Explore experiences of carers supporting women with IDs during breast screening	Qualitative	Carers ensure breast health, varied abilities in breast checks, communication challenges; barriers include time, pain, and fear	Caregiver support is crucial; policy changes are needed to improve access	****
[37] UK	Assess need for targeted breast cancer awareness intervention for women with mild/moderate IDs	Qualitative	Low health focus, varied health consciousness, poor caregiver awareness, need for accessible resources	Combined health and breast awareness intervention needed; engage caregivers and HCPs	*****
[38] USA	Identify barriers and opportunities for improving cancer screening in adults with IDD via nurse survey	Qualitative	Lower screening rates, undetected cancer cases, various screening barriers	Tailored approaches needed; identify successful models for broader implementation	****
[29] UK	Examine how community nurses and residential staff support women with IDs in breast screening	Qualitative	Nurse support for appointments, limited knowledge of breast cancer, barriers to attendance	Disparities in access due to lack of educational materials, need for better health promotion	****
[39] UK	Investigate knowledge, attitudes, and decision-making in cancer screening for women with LDs, involving carers	Mixed design	Carer input crucial, limited awareness of cancer symptoms, need for accessible materials	Easy-to-read documentation and inclusive approaches essential	****
[40] USA	Explore family caregivers’ perspectives on cervical and breast cancer screening for women with IDs	Qualitative	Caregiver beliefs influence decisions, emotional and practical barriers	Include caregivers in health promotion; address preparation and relaxation	*****
[3] UK	Investigate healthcare professionals’ perspectives on supporting women with IDs in breast cancer screening	Qualitative	Practice nurses advocate for equal access, challenges in self-examination, GP encouragement	Interdisciplinary collaboration needed for equitable screening access	***
[41] Ireland	Assess nurses’ proficiency, motivation, and knowledge on breast cancer screening in ID settings	Quantitative	Uncertainty in detecting anomalies, knowledge deficits on risk factors	Comprehensive strategies needed to address training and knowledge gaps	***
[21] UK	Investigate care staff involvement in cancer prevention for individuals with IDs	Qualitative	Minimal training received, lack of awareness of cancer risk factors	Improved education and collaboration with cancer professionals needed	*****
[42] Canada	Examine family members’ views on preventive healthcare for relatives with IDs	Qualitative	Decision-making influenced by family dynamics, need for tailored educational approaches	Educational interventions should address family dynamics	****
[43] Canada	Explore primary care providers’ experiences in recommending cancer screening to patients with IDs	Qualitative	Equal care emphasis, individualized care approach	Further research needed on family physicians’ perspectives	***
[44] USA	Customize educational program for Native American women with IDD	Qualitative	Financial barriers, communication challenges, cultural considerations	Adapt educational programs for cultural contexts	****
[12] USA	Investigate barriers to mammogram participation among individuals with IDs	Qualitative	Caregiver challenges, lack of education, physical inaccessibility	Improve care coordination; address anxiety and accessibility issues	****

(***: 60% quality, **** 80% quality, ***** 100% quality)

## 3. Results

### 3.1. Search Outcome

**Figure 1:**
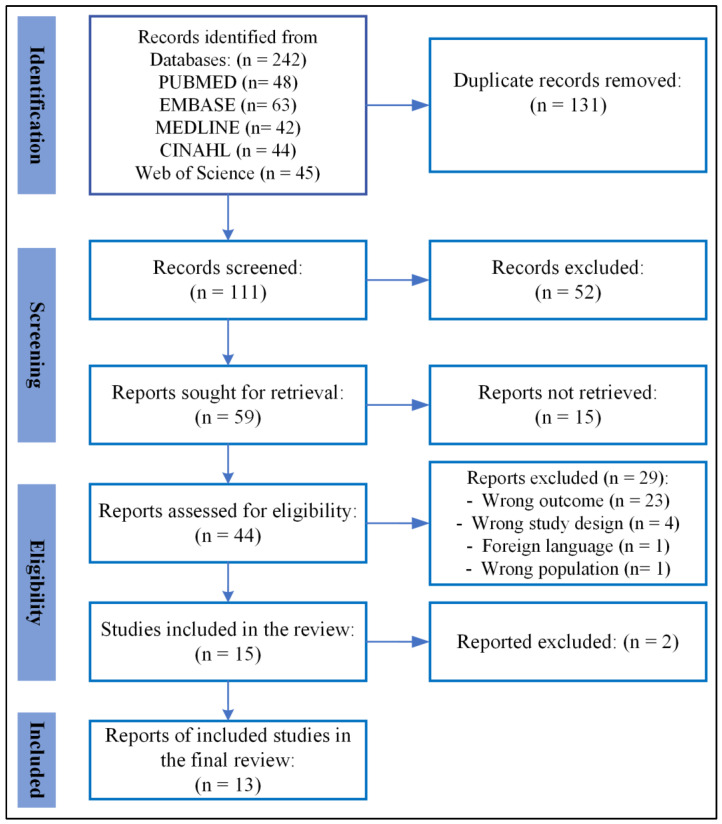
PRISMA flowchart illustrating the results of the literature search and screening procedure for the most recent studies on the perspectives of caregivers regarding cancer prevention among individuals with intellectual disability.

The search was conducted across five databases, resulting in the identification of 242 articles through the application of title, abstract, and English-language filters. After eliminating 131 duplicate articles using Endnote, the remaining 111 records underwent a meticulous assessment of eligibility criteria. Following a comprehensive peer evaluation of the search strategy, the titles and abstracts of these records were screened, leading to the exclusion of 52 articles. Subsequently, the remaining 59 articles underwent a thorough full-text review and evaluation against eligibility criteria. Before full-text screening, 15 articles were not retrieved, thus meaning that only 44 articles underwent full-text screening. After the comprehensive review of full-text articles, 29 articles were excluded. Among these exclusions, 23 were due to incorrect outcomes, 4 were due to an inappropriate study design, 1 article was excluded due to the wrong study population, and 1 was excluded as it was composed in a foreign language. Out of the 15 studies encompassed within this review, only 13 were ultimately incorporated and discussed in the present analysis. The details of the selection process are presented in the PRISMA flow diagram (Figure 1).

### 3.2. Study Characteristics

The thirteen studies selected span a variety of geographical locations, including the United Kingdom (UK) [3,21,29,37,39], the United States (USA) [12,38,40,44], Canada [42,43], Scotland [36], and Ireland [41]. 

The studies employ various study designs, including qualitative approaches [3,12,21,29,36,37,38,40,42,43,44], one quantitative study [41], and one mixed-design study [39]. 

The studies collectively explore perspectives on cancer prevention in adults with intellectual disabilities, primarily focusing on breast cancer screening. They involve a spectrum of participants, including healthcare professionals, caregivers (both paid and family), individuals with intellectual disabilities, and family members. The thematic synthesis of these studies provides valuable insights into the roles, knowledge, challenges, and decision-making processes relevant to cancer prevention within different caregiving and healthcare settings.

### 3.3. Thematic Synthesis

The thematic synthesis utilized Thomas and Harden’s (2008) [35] approach to analyze caregiver perspectives on cancer prevention for adults with intellectual disabilities in both institutional and home care settings. This systematic process allowed for the identification of five key themes, each reflecting the roles and challenges caregivers faced.

- Theme 1: Caregivers’ Perspectives

Caregivers, particularly nurses, recognize significant disparities in cancer screening for individuals with intellectual disabilities (IDs) [3,38,43]. These disparities are often attributed to various barriers such as the need for sedation, communication difficulties, and the absence of tailored healthcare protocols. Consequently, individuals with IDs receive fewer cancer screenings compared to the general population [38].

To address these gaps, healthcare providers advocate for interventions that include education and training for staff, modifications to procedures, and improved accessibility. Nurses, in particular, report that primary care clinicians often fail to adapt screening procedures to meet the needs of individuals with IDs. Such failures further contribute to the disparities seen in cancer screening rates for this population.

Healthcare professionals also emphasize the importance of adopting a comprehensive, individualized approach to screening. This includes addressing the unique physical and emotional challenges that individuals with ID face during these procedures, such as discomfort, literacy issues, and difficulties in providing informed consent. Tailored healthcare protocols, along with psycho-social support, are viewed as essential strategies for improving access to cancer prevention services [3].

Several studies highlight the need for increased awareness and interdisciplinary collaboration among healthcare providers and caregivers to overcome these barriers. For example, the CUPID project demonstrates the effectiveness of involving various stakeholders, including self-advocates and support professionals, in the development of policy and adaptations to healthcare practices aimed at reducing disparities in cancer prevention for people with IDs [45]. Initiatives like these underscore the importance of stakeholder involvement and the need for continuous education and training [1].

- Theme 2: Breast Cancer Screening Perspectives

Caregivers play a pivotal role in supporting women with intellectual disabilities (IDs) during breast cancer screening, helping them navigate the multiple challenges that arise in this process. A key challenge is explaining the screening procedure to women with IDs, which is often compounded by limited awareness, negative emotions, and practical barriers, such as discomfort during the screening itself [3,12,29,36,42,43]. These challenges contribute to lower participation rates in breast cancer screening programs for women with IDs compared to the general population [40].

Caregivers, both family members and professional staff, are essential in preparing women with IDs for breast cancer screenings by offering emotional support, mitigating anxiety, and addressing privacy concerns. Their involvement is critical to overcoming logistical barriers and discomfort during the procedure, which are common deterrents to participation [3,46]. The support provided by caregivers can significantly impact whether women with IDs engage in these screening programs, highlighting their indispensable role in ensuring access to necessary health services.

Disparities in access to breast cancer screening for women with IDs are often linked to the level of caregiver support and knowledge. Many caregivers report a lack of tailored educational resources to guide them through the process of supporting women during breast cancer screenings. This gap in resources underscores the need for targeted educational interventions that equip caregivers with the skills and knowledge required to address the unique needs of women with IDs. Such interventions would help promote inclusivity and improve access to essential health services, reducing the disparities in breast cancer screening participation [47,48].

- Theme 3: Caregivers’ Knowledge and Barriers to Cancer Prevention

Caregivers, including healthcare professionals, often exhibit limited knowledge about cancer prevention for individuals with intellectual disabilities (ID), highlighting the need for tailored educational interventions [21,37,38,41]. Suggested approaches include easy-to-read materials and integrated healthy living and breast awareness programs specifically designed for people with IDs. The lack of comprehensive training among residential care staff further underscores the importance of enhancing education in health promotion activities.

Barriers to cancer screening in both institutional and home care settings are significant. These include fear, discomfort, a lack of awareness, and logistical challenges. Additionally, caregiver beliefs, women’s comfort levels, and decision-making processes play crucial roles in determining whether individuals with IDs undergo screening [3,12,21,39,40,41,43]. Financial concerns, hesitancy, and resistance to screening are also common, further emphasizing the need for culturally sensitive care approaches. Overwhelming caregiver responsibilities, fatigue, a lack of health education, and missed opportunities contribute to these barriers, making it even more challenging to ensure regular cancer screening participation.

Limited access to user-friendly, accessible information—especially for adolescents and young adults—is another key barrier. Family carers and healthcare professionals play a crucial role in overcoming these challenges, particularly in home care settings, where carers are the primary source of support [49].

In institutional care, patients benefit from structured support systems such as supervised living arrangements and regular healthcare services. However, pervasive issues like fear, distress, and communication difficulties often hinder screening. These barriers are compounded by negative interactions with healthcare professionals or the absence of services tailored to the specific needs of individuals with IDs. As a result, adults with IDs frequently experience significant distress during cancer screening, emphasizing the need for individualized approaches to alleviate discomfort [50].

To improve access to screening, evidence-based guidelines and targeted interventions must be developed to address these unique challenges in both institutional and home care settings. These efforts should focus on increasing accessibility by reducing scheduling difficulties and adapting communication strategies [51]. Additionally, healthcare professionals need training to recognize and respond to the specific cancer risks faced by individuals with IDs, ensuring that cancer screening becomes a routine part of their care [52].

Despite the barriers, both institutional and home care settings offer opportunities to enhance screening participation. In institutional environments, structured healthcare services can empower individuals with IDs, while in home care, strong relationships with caregivers can encourage engagement with screening programs.

- Theme 4: Cultural Context and Decision-Making

Cultural context plays a crucial role in shaping decision-making around cancer screening for individuals with intellectual disabilities (IDs) [12,43,44]. Family caregivers and healthcare providers are key mediators in this process, balancing factors such as quality of life, fear of distress, and cultural values. Decision-making in this context emphasizes the importance of individualized care that considers not only intellectual disability but also broader socio-cultural factors.

## 4. Discussion

The exploration of caregiver perspectives on cancer prevention for adults with intellectual disabilities in both institutional and home care settings reveals intricate challenges and opportunities within the healthcare landscape. This discussion delves into the key themes that emerged from the literature review and their implications for practice, policy, and future research.

Identified challenges in explaining breast cancer screening to individuals with intellectual disabilities underscore the need for targeted educational interventions. Caregivers, including healthcare professionals, play a pivotal role in ensuring that women with intellectual disabilities receive the necessary support during the screening process. Disparities in access highlight the importance of tailoring health education materials and interventions to address the unique needs and considerations of individuals with intellectual disabilities and their families.

Caregiver involvement emerges as a crucial factor in cancer services, with an emphasis on considering the entire family and providing emotional support [53]. Regular cancer screening programs are deemed essential, given the increased prevalence rates among adults with intellectual disabilities [52]. However, the data also highlights disparities in healthcare access, with concerns about late cancer diagnoses [38,54]. Specialized care tailored to the needs of this population is deemed essential [55]. General practitioners (GPs) play a crucial role in providing proactive support following cancer diagnosis, addressing communication, capacity, and consent issues [54]. This underscores the importance of caregiver involvement in developing tailored cancer services [49]. The limited knowledge demonstrated by caregivers and healthcare professionals regarding cancer prevention and awareness signals a critical gap in training and education. The recommendation for tailored educational approaches and the emphasis on enhancing education for residential care staff emphasize the necessity for comprehensive training programs. These programs should not only focus on increasing knowledge but also on building the skills necessary to support individuals with intellectual disabilities effectively. Despite these identified needs, there is a substantial dearth of services and appropriate cancer information for people with intellectual disabilities [2]. This underscores the urgency to address obstacles and barriers to high-quality cancer care for this vulnerable population [1,26].

Identified barriers to cancer screening, including fear, discomfort, and logistical challenges, highlight the complex decision-making processes faced by caregivers and individuals with intellectual disabilities. Financial concerns and hesitancy toward screenings add layers of complexity, necessitating targeted interventions to address these specific challenges. The findings emphasize the importance of considering the holistic well-being of individuals with intellectual disabilities, including addressing fatigue and overwhelming responsibilities.

The influence of cultural context on decision-making highlights the importance of culturally sensitive care. Adapting educational programs to the cultural context and respecting the preferences of individuals and their families are crucial steps toward improving engagement in cancer prevention activities. For example, in some cultural settings, caregivers and healthcare professionals may avoid disclosing cancer diagnoses or treatment options to individuals with IDs, fearing that such information might cause emotional distress. However, this protective approach can inadvertently limit the individuals’ participation in decision-making, leading to underinformed choices. Tailored communication strategies and culturally sensitive models for breaking bad news are essential to improving engagement and outcomes for this population [56].

Family caregivers, central to the decision-making process, often prioritize their loved ones’ overall well-being and quality of life. In many cases, caregivers weigh the potential benefits of cancer screening against the emotional and physical impacts of the procedures, sometimes choosing comfort and emotional stability over preventive screenings. Cultural and familial values frequently influence these decisions, where maintaining comfort may take precedence over invasive preventive measures [42].

Healthcare providers also recognize the need to adapt educational programs and decision-making processes to fit the cultural contexts of specific populations. Ensuring that communication is culturally sensitive and that support systems align with patients’ needs is crucial for fostering effective shared decision-making [57]. Providers highlight the importance of culturally relevant models that engage both caregivers and individuals with IDs in making informed decisions about cancer screening and overall health care.

The data also highlight the multifaceted challenges and critical considerations surrounding caregiver engagement in cancer prevention and health promotion activities for individuals with intellectual disabilities (IDs). The recognition of early signs and symptoms is challenging due to cognitive impairment and communication issues [21]. Advocacy is identified as crucial to ensuring appropriate healthcare and health promotion for adults with intellectual disabilities, especially in the context of cancer care [55]. Accessible informational materials are emphasized as crucial, pointing out the necessity for further development to address the cancer-related needs of people with intellectual disabilities [53]. Disparities in secondary prevention are noted, emphasizing the critical role of secondary prevention in a population that may struggle to recognize early signs and symptoms [58]. Caregiver involvement in decision-making within cancer care is highlighted, noting that communication and decision-making are often mediated through support workers or family carers [17]. Accessible consent in the context of cancer care for adults with intellectual disabilities is emphasized [49]. The importance of experience and training is noted, with previous experience working with ID patient groups enhancing caregivers’ confidence in communication and providing appropriate care [59]. Emotional and social support are recognized as crucial in caregiving and coping with challenging situations [53]. The data also underline the need for unified national and local policies to reduce health inequalities and promote the inclusion of people with intellectual disabilities in cancer prevention and screening measures [1]. The importance of raising public awareness about the challenges faced by people with intellectual disabilities concerning cancer prevention is also emphasized [1].

Moreover, the data highlight disparities in cancer screening for individuals with intellectual disabilities [60]. The timeliness of cancer diagnosis and treatment for people with intellectual disabilities poses a significant challenge, with limited evidence suggesting differential treatment and higher cancer-related mortality in this population [1,60]. Overall, the data call for a comprehensive and inclusive approach, addressing communication barriers, providing caregiver support, advocating for policy changes, and enhancing public awareness to improve cancer outcomes for individuals with intellectual disabilities. The recognition of disparities in cancer screening for individuals with intellectual disabilities by healthcare professionals underscores the need for systemic changes. Proposed interventions, such as education, training, and improvements in accessibility, point toward a multifaceted approach that involves healthcare professionals, caregivers, policymakers, and individuals with intellectual disabilities themselves. Collaborative efforts are essential to bridge the existing gaps and promote equitable access to cancer screening services.

The World Health Organization (WHO) has recognized the challenges in addressing cancer prevention among people with intellectual disabilities, emphasizing policies that promote equity and access to healthcare services. Research consistently highlights the importance of tailored approaches to ensure that this vulnerable population is not excluded from cancer prevention initiatives.

In the European Union (EU), there is a growing awareness of the need to address cancer prevention for people with intellectual disabilities. The CUPID (Cancer-Understanding Prevention in Intellectual Disabilities) project stands at the forefront of these efforts, aiming to raise awareness, develop tailored policy recommendations, and establish a network of stakeholders to combat health inequalities. The project underscores the importance of unifying national and local policies to ensure that cancer prevention and screening measures are inclusive and responsive to the specific needs of this population [1,45].

One of the significant challenges identified is the higher likelihood of late-stage cancer diagnoses among individuals with intellectual or developmental disabilities, particularly in breast and colorectal cancers. This disparity underscores the need for targeted cancer prevention strategies within EU policies to address these inequities and ensure timely diagnosis and treatment [61].

Additionally, EU policies are increasingly focusing on the ethical adjustments required in healthcare services to ensure that people with intellectual disabilities have access to inclusive and accessible cancer care. This includes training healthcare professionals to adapt their approaches and integrating ethical considerations into treatment decisions to enhance the quality of care for this population [62].

By involving diverse stakeholders across Europe, the CUPID initiative aims to identify necessary actions over the next four years to improve cancer prevention and response services for individuals with intellectual disabilities. It ultimately strives to enhance equity compared to the general population [45].

In summary, the synthesized data underscore the pressing need for accessible cancer information tailored to individuals with intellectual disabilities. Various studies advocate for the proactive provision of user-friendly information, addressing the unique requirements of this population [2,49]. Additionally, staff training is identified as crucial to enhancing knowledge about early cancer signs and symptoms [21]. This aligns with the findings of [40], emphasizing that preparation before exams is a significant enabler for women with intellectual disabilities to receive cancer screening.

### 4.1. Implications for Practice and Policy

This synthesis of caregiver perspectives on cancer prevention emphasizes the need for targeted educational interventions and comprehensive training programs for caregivers and healthcare professionals. The implications for practice and policy highlight the importance of enhancing caregiver education and support, as caregivers play a crucial role in facilitating cancer screening for individuals with intellectual disabilities (IDs). Future research should focus on developing tailored educational resources that equip caregivers with practical tools and ongoing training in cancer prevention and screening protocols. Additionally, healthcare providers must adopt individualized and culturally sensitive care approaches, adapting cancer screening procedures and communication strategies to meet the unique needs of individuals with IDs. This requires the exploration of culturally relevant models of care and effective engagement in shared decision-making, supported by policies that prioritize cultural competence. Addressing barriers to screening necessitates healthcare system adaptations, such as implementing disability-friendly protocols and incorporating psycho-social support into routine care. Furthermore, promoting collaborative decision-making involves integrating caregivers into the healthcare planning process, ensuring that decisions consider both the health needs of individuals with IDs and their overall well-being. Finally, reducing disparities in cancer prevention services calls for targeted interventions, such as community outreach programs and mobile screening units, backed by policy reforms that guarantee equitable access and allocate resources to improve the inclusion of individuals with IDs in national cancer prevention initiatives. Practices should be tailored to the cultural context, and policies should prioritize inclusivity, accessibility, and support for caregivers.

### 4.2. Limitations

While this review provides a comprehensive overview of the existing literature on the perspectives of caregivers regarding cancer prevention among individuals with intellectual disabilities, it is important to acknowledge several limitations. First, the potential for publication bias cannot be ignored, as this review relies on published studies and may miss relevant unpublished research, which could provide additional insights into caregiver perspectives. Additionally, the included studies exhibit varying levels of methodological quality, which may introduce bias and affect the overall robustness of the findings. This variability impacts the reliability of the results and their applicability in practice.

The heterogeneity among studies complicates the synthesis of results and may limit the ability to draw comprehensive conclusions regarding best practices for improving accessibility and education in cancer prevention. For example, while some studies may have demonstrated effective educational interventions, their applicability may vary significantly across different cultural contexts. Thus, the practical implications derived from this review must be considered with caution, as the findings may not uniformly translate into effective strategies for diverse populations.

Moreover, the studies cover diverse geographical locations, which may limit the generalizability of findings to all regions or cultural contexts. Specific examples of how interventions can be tailored to various cultural settings, such as using culturally relevant communication strategies or engaging community leaders to promote cancer awareness, would strengthen this aspect of the review. Additionally, the temporal variation in the publication years of the studies may not fully capture changes in societal attitudes, healthcare policies, or interventions over time, further complicating the applicability of the findings.

The review’s focus primarily centers on breast cancer screening, which may overlook other aspects of cancer prevention or different types of cancer that have unique considerations and may require distinct approaches. Lastly, due to practical constraints, this review was limited to studies published in the English language, potentially introducing language bias and excluding important articles in other languages. This limitation emphasizes the need for future research to explore a broader range of studies, including those published in other languages, to capture a more comprehensive understanding of caregiver perspectives across diverse cultural contexts.

## 5. Conclusions

In conclusion, this literature review provides valuable insights into the challenges and opportunities in cancer prevention for adults with intellectual disabilities (IDs) and emphasizes the critical role that caregivers play in supporting individuals during cancer screening processes. The synthesis highlights the multifaceted challenges caregivers face, including barriers related to communication, emotional support, and cultural considerations. Understanding and addressing caregiver perspectives are essential steps toward developing holistic and inclusive strategies for cancer prevention.

Future research should focus on several key areas to address the identified gaps. First, studies should investigate the effectiveness of tailored educational interventions designed specifically for caregivers, assessing how these programs can enhance their knowledge and skills in supporting individuals with IDs through the screening process. Second, research should explore innovative methods for improving access to cancer-related information, ensuring that it is user-friendly and culturally relevant for both individuals with IDs and their caregivers. This could involve developing accessible digital resources or community outreach initiatives. Additionally, future studies should examine the impact of cultural competence training on healthcare professionals’ perspectives, with the aim of fostering a more inclusive healthcare environment.

By recognizing the importance of these targeted research efforts, we can enhance the effectiveness of cancer screening and prevention initiatives for individuals with IDs. Furthermore, addressing systemic disparities in access to screening services is vital for promoting equitable healthcare outcomes. The findings of this review contribute to the growing body of knowledge in this area, paving the way for improved practices, policies, and interventions that prioritize the unique needs of this population. Ultimately, prioritizing inclusivity and accessibility in both practice and policy will be crucial in bridging the gaps in cancer prevention and ensuring that all individuals receive the quality care they deserve.

## Data Availability

The data supporting the findings of this study are available within the article.

## Appendix A

Full Search Strategy Used in Article Titles and Abstract Search.

**Search Component**	**Keywords**
Caregiver	“Caregiver” OR “Carers” OR “Carer” OR “Care Givers” OR “Care Giver” OR “Spouse Caregivers” OR “Family Caregivers” OR “Informal Caregivers” OR “Caretakers” OR “Guardians” OR “Providers” OR “Nurses” OR “Healthcare providers” OR “Medical caregivers” OR “Patient care providers” OR “Health attendants” OR “Healthcare professionals”
Cancer Prevention	“Cancer prevention” OR “preventive measures” OR “health promotion” OR “risk reduction” OR “knowledge levels” OR “awareness” OR “understanding” OR “information” OR “viewpoints” OR “attitudes” OR “beliefs” OR “experiences” OR “health education” OR “behavioral change” OR “screening practices” OR “early detection” OR “health communication” OR “intervention strategies” OR “public health initiatives” OR “health literacy”
Intellectual Disabilities	“Intellectual Disabilities” OR “Mental Retardation” OR “Intellectual Development Disorder” OR “Development Disorder” OR “Development Disorders” OR “Mental Retardation” OR “Intellectual Disability” OR “Idiocy” OR “Mental Retardation” OR “Mental Retardations” OR “Mental Deficiencies” OR “Mental Deficiency” OR “Learning Disabilities” OR “Cognitive Impairments” OR “Developmental Disabilities” OR “Cognitive Challenges” OR “Intellectual Challenges” OR “Cognitive Disorders”
Residential Care	“Residential care” OR “Facility-based care” OR “Care in institutions” OR “Institutional living” OR “Group care” OR “Long-term care” OR “Nursing home care” OR “Assisted Living” OR “Facility care” OR “Institutional care” OR “home care” OR “caregiving environments”

## Appendix B

Full Data Extracted From the Reported Articles.

**Author, Year, Country**	**Aim**	**Type of Study**	**Key Findings**	**Conclusion**	**Quality**
[36] Scotland	Investigate the perspectives and experiences of paid and family carers in supporting women with intellectual disabilities during breast screening.	Qualitative	Active Caregiver Involvement: Carers actively ensured the breast health of women with intellectual disabilities. Dichotomy in Abilities: Recognition of varying abilities among women in undertaking breast checks themselves. Need for Assistance: Most carers acknowledged the need for assistance, highlighting unique challenges. Communication Challenges: Difficulties in explaining breast cancer screening, symbolized by the metaphor, “It could be broccoli.” Universal Support: Decision-makers unanimously believed women with intellectual disabilities should be offered breast screening. Obstacles Reported: The main barriers included limited time, pain, and fear, with external factors influencing accessibility to screening.	Caregiver reassurance is vital for women’s support, emphasizing the need for improved working relationships with screening staff. Policymakers should extend screening to community levels, addressing resource challenges. Tensions persist between encouraging attendance and resource limitations. Varied support needs depend on women’s intellectual abilities and caregivers. Findings indicate potential shortcomings across the breast screening process.	****
[37] UK	Examine the necessity for a targeted breast cancer awareness intervention for women with mild/moderate intellectual disabilities, gathering insights into preferred intervention processes and content.	Qualitative	Low Focus on Health: Individuals with intellectual disabilities (IDs) may have jobs and income, but their attention to diet, lifestyle, and exercise is generally low. Varied Health Consciousness: Some healthcare professionals acknowledged that individuals with IDs can be health-conscious, especially regarding activities like walking and jogging. Lack of Education and Support: Caregivers and healthcare professionals lack educational, training, and support needs for breast cancer awareness in women with IDs. Poor Caregiver Awareness: Healthcare professionals noted caregivers’ poor awareness levels, similar to the general population, regarding breast cancer risk, outcomes, prognosis, and treatment. Need for Accessible Resources: Caregivers emphasized the absence of easy-to-read formats and educational resources specific to breast cancer awareness for women with IDs. Limited Knowledge: Overall, caregivers demonstrated limited knowledge about breast cancer.	- A combined healthy living and breast awareness educational intervention is recommended for women with mild/moderate levels of intellectual disability (ID). - Engaging caregivers and healthcare professionals (HCPs) in education and training is crucial.	*****
[38] USA	Understand barriers and opportunities for improving cancer screening in adults with intellectual and developmental disabilities through a survey of nurses working in developmental disability settings.	Qualitative	Perceived Disparities: Nurses perceive lower cancer screening rates among adults with intellectual and developmental disabilities. Instances of Undetected Cancer: Half of the nurses encountered cases where cancer developed without prior screening for individuals with intellectual and developmental disabilities. Barriers to Successful Screenings: Various reasons for unsuccessful cancer screenings include lack of cooperation, test completion issues, screenings not ordered during preventive visits, fear, refusal, and lack of understanding. Proposed Interventions: Nurses suggest interventions encompassing education, training, accessibility improvements, financial support, procedure modifications, and tracking measures for enhanced cancer screening.	**Identifying Successful Models:** Acknowledges the potential existence of successful cancer screening models for individuals with disabilities, urging further identification, study, and replication for broader implementation. **Inclusive Perspectives:** Highlights the importance of diverse perspectives from self-advocates, direct support professionals, hospital technicians, and physicians for a comprehensive understanding of factors contributing to disparities in cancer screening for persons with disabilities. **Tailored Approaches:** Nurses emphasize the need for tailored approaches addressing educational, logistical, and personal barriers to cancer screening for individuals with disabilities.	****
[29] UK	Explore how community nurses and residential staff support women with intellectual disabilities in accessing breast screening services.	Qualitative	Nurse Support: Community intellectual disability nurses (CIDNs) assist women with intellectual disabilities (IDs) in attending medical appointments, including breast screening, by providing transport and accompanying them for mammograms. Limited Knowledge: Women with IDs may have limited knowledge about breast cancer due to poor literacy, communication, and understanding. Barriers to Attendance: Negative emotions and attitudes, such as privacy concerns, fear, discomfort, practical issues (distance, cost, wheelchair access), and other health problems contribute to refusals to attend breast screening. Awareness Challenges: Women, family carers, and residential staff are informed about breast screening appointments only after receiving invitation letters. Some family carers may decide not to inform their daughters/siblings, citing various reasons such as priority, taboo, or inappropriateness.	- There are disparities in access to breast screening. - Disparities in access are attributed to a lack of appropriate health educational material. - Health promotional activities are insufficient, contributing to the disparities. - There continues to be a lack of understanding among women with IDs regarding what to expect and the potential consequences of screening. - This format summarizes the key points from the conclusion, focusing on the disparities in access to breast screening and the contributing factors highlighted in the study.	****
[39] UK	Investigate the knowledge, attitudes, and decision-making processes surrounding cervical and breast cancer screening in women with learning disabilities, involving input from family and paid carers.	Mixed design	- A paid carer emphasized that sexual status should not determine eligibility, as underlying issues may exist. - Family carers noted a reliance on them to explain screening-related information to women with learning disabilities upon receiving screening letters. - Participants expressed concerns about women with learning disabilities having limited awareness of cancer symptoms and believed they might not be informed due to societal hesitance to discuss such issues. - Suggestions for enhancing screening experiences included sharing positive experiences, ensuring healthcare professionals are informed about women with learning disabilities’ needs, utilizing accessible materials like passport books, and actively involving women with learning disabilities in discussions about making the screening process more manageable.	- Implementing easy-to-read documentation throughout the screening process is crucial to facilitate understanding. - Actively inviting women with learning disabilities to attend cancer screening helps promote inclusivity and accessibility. - Emphasizing awareness of cancer symptoms among women with learning disabilities is essential for informed decision-making. - Ensuring carers are well-informed and supportive of women with learning disabilities decisions is a key factor in the screening process. - Recognizing the need for reasonable adjustments at every stage of the cancer screening pathway, from invitation to result communication, is essential for providing effective care to women with learning disabilities.	****
[40] USA	Explore family caregivers’ perspectives on various aspects of cervical and breast cancer screening for women with intellectual disabilities.	Qualitative	Caregiver Beliefs: Caregiver beliefs significantly influence decision-making, with some deeming breast exams unnecessary. History of Sexual Abuse: Women with intellectual disabilities may feel uncomfortable due to a history of sexual abuse. Doctor’s Gender and Familiarity: The gender of the doctor and the woman’s familiarity with them impact decision-making. Clear Explanations: Clear explanations from caregivers and doctors positively influence decision-making. Emotional Factors: Embarrassment, shyness, and fear contribute to decision-making, affecting comfort levels. Sexual Activity and Pain Perception: Women not sexually active or perceiving the exam as painful may choose to avoid it. Caregiver Consent and Support: Caregiver consent and support, including staying in the exam room, influence decisions. Insurance Coverage: Insurance coverage, ability to pay, and changes in coverage impact decision-making. Medical History: A history of hysterectomy or specific medical conditions may affect the perceived necessity of the exam. Anti-Anxiety Medications: The use of anti-anxiety medications may influence the decision to undergo the exam. Transportation: The cost and availability of transportation contribute to decision-making.	- Family caregivers emphasized the significance of adequate preparation, relaxation, and their own participation in the examination room to facilitate cervical and breast cancer screening for their loved ones with intellectual disabilities. - Preliminary evidence suggests that family caregivers often lack sufficient knowledge regarding the necessity of cervical and breast cancer screening for women with intellectual disabilities. - Efforts must be directed toward including family caregivers in health promotion interventions aiming to increase cervical and breast cancer screening for women with intellectual disabilities.	*****
[3] UK	Investigate healthcare professionals’ perspectives on their roles in supporting women with intellectual disabilities in accessing breast cancer screening.	Qualitative	Practice Nurses’ Advocacy: Practice nurses emphasize equal access to health screening for women with intellectual disabilities, advocating against discrimination. Challenges in Self-Examination: Breast care nurses highlight challenges in self-examination for women with intellectual disabilities due to cognitive deficits and communication barriers. GP Encouragement: GPs use opportunistic encounters to encourage breast screening, often collaborating with the community intellectual disability team or family. Roles of Breast Care Nurses: Screening unit breast care nurses explain the screening process, offer health promotion, and provide post-screening or breast cancer diagnosis support. Breast Cancer Risk Factors: Primary healthcare staff identify various risk factors, including literacy, exercise, family history, and environmental factors. Importance of Carer Support: Lack of carer support is acknowledged as a potential barrier, highlighting the need for accompaniment to appointments. Professional Attitudes as Barriers: Healthcare professional attitudes and experience, including misconceptions about screening appropriateness, may serve as barriers.	A comprehensive approach involving heightened awareness, continuous education, and interdisciplinary collaboration is pivotal to break down the barriers hindering women with intellectual disabilities from accessing crucial breast cancer screening services. By reinforcing the roles of healthcare professionals and fostering collaborative efforts, we can work toward achieving equitable breast cancer prevention and control for all women, irrespective of intellectual ability.	***
[41] Ireland	Explore the proficiency, motivation, and knowledge levels related to breast cancer screening and awareness among nurses working in intellectual disability settings.	Quantitative	- Nurses expressed uncertainty about their proficiency in detecting personal breast anomalies. - Participants’ motivation for engaging in personal breast self-examination (BSE) and their self-perceived susceptibility to breast cancer was measured. - A significant majority of nurses considered BSE valuable for detecting breast lumps/changes themselves. - Nurses demonstrated a knowledge deficit regarding specific breast cancer risk factors, as evidenced by a portion of nurses disagreeing or being unsure about factors such as the incidence of breast cancer with age, the impact of childbirth, and menopause-related factors.	The study reveals deficiencies in supporting breast awareness initiatives for women with intellectual disabilities, exposing knowledge and skill deficits among nurses. These findings indicate potential gaps in nursing professionals’ training and preparation, extending beyond the intellectual disability perspective. The study underscores broader concerns and challenges faced by nurses in promoting breast awareness. Emphasizing the need for comprehensive strategies, the conclusion calls for systemic interventions to address these challenges at a broader level.	***
[21] UK	Investigate how care staff actively participate in cancer prevention and health promotion activities for individuals with intellectual disabilities.	Qualitative	- Only a small fraction of staff members reported receiving any form of training related to cancer awareness and health promotion activities designed for individuals with intellectual disabilities. - Further exploration revealed that the training received by the few staff members was minimal, and in some instances, it only consisted of passing on written information to the staff. - The lack of formal and comprehensive training indicates potential gaps in the preparation of residential care staff to address cancer-related issues in individuals with intellectual disabilities. - Gaps were identified not only in general cancer awareness but also specifically in understanding the signs, symptoms, and risk factors associated with cancers such as stomach, breast, cervical, and prostate cancer. - Respondents demonstrated a lack of awareness regarding both the risk factors that contribute to cancer development and the protective factors that may mitigate the risk. - The majority of respondents had not received training in health promotion tailored for individuals with intellectual disabilities.	- Individuals with intellectual disabilities deserve equal access to cancer prevention services, necessitating tailored strategies for their unique needs. - There is a clear need for improved education and training among staff, focusing on raising awareness of cancer signs, symptoms, risk factors, and protective measures for individuals with intellectual disabilities. - Collaboration between intellectual disability and cancer professionals is vital to developing effective, accessible health promotion strategies.	*****
[42] Canada	Examine family members’ views on preventive healthcare decision-making and their perceptions of quality healthcare for relatives, and discern barriers and facilitators of mammography.	Qualitative	- Some participants associated mammography with challenges related to their loved one’s sexuality, impacting the decision-making process. - Mammography was seen as a means for women with intellectual disabilities to connect with a larger community of women, emphasizing a sense of belonging. - The decision to undergo mammography held significant meaning, with participants viewing it as a civil rights issue and emphasizing the need for equal opportunities. - Decision-making focused on preserving the quality of life, avoiding suffering, and prioritizing comfort over dramatic interventions. - Past medical scares influenced decision-making, reflecting a desire to prioritize the well-being of their loved ones. - Fears related to potential cancer diagnoses and concerns about the impact on quality of life were prominent among participants. - Additional support, including time, detailed explanations, facility tours, sedation, and family presence, was deemed necessary for successful mammograms. - Participants emphasized the need for tailored educational approaches based on their loved one’s learning style.	- Cancer screening decisions for women with IDs are intricately linked to family dynamics, emphasizing the complexity of the decision-making process. - Disparities in mammography access for women with IDs within families underscore the need to address and understand family dynamics in intervention strategies. - Obtaining a mammogram involves multi-level preparation and family deliberation, requiring interventions that acknowledge and appreciate these complexities. - There is a significant need for educational interventions targeting families, tailored to address the unique considerations involved in mammography decision-making within families with members with IDs.	****
[43] Canada	Investigate the experiences of primary care providers in recommending cancer screening to patients with intellectual disabilities.	Qualitative	Primary care providers view intellectual disability as one of several factors influencing cancer screening recommendations. They emphasize equal care for all, considering various patient characteristics holistically. Despite evolving guidelines, clinicians’ express commitment to individualized care based on patient needs.	Physicians with favorable attitudes toward intellectual disability are more likely to recommend cancer screening. A nuanced approach balancing guidelines and patient interests characterizes the decision-making process. The study emphasizes the complexity physicians face in recommending preventive measures and calls for further research to explore experienced family physicians’ perspectives on routine cancer screening.	***
[44] USA	Collect data for customizing educational programs to fit the cultural context of Native American women with IDD.	Qualitative	Financial barriers impact cancer care for women with IDD. Hesitancy stems from fear and trauma, while a lack of health education contributes to misinformation. Communication challenges underscore the need for tailored care, respecting preferences and cultural considerations. Legal concerns and room design also influence the healthcare experience for women with IDD.	The conclusion emphasizes the use of an essential elements approach to guide the adaptation of the original cancer screening education program for Native American women with IDD in urban and rural contexts.	****
[12] USA	Investigate barriers and facilitators associated with mammogram participation among individuals with intellectual disabilities.	Qualitative	- Caregivers recognize mammogram importance but face challenges in managing overwhelming responsibilities. - Fatigue and care duties sometimes hinder caregivers from supporting mammogram appointments. - Care coordination support is beneficial, aligning with Pennsylvania 6400 regulations. - A lack of health education, especially for those attending adult day programs for extended hours. - Missed opportunities in social settings for healthcare discussions and education.	- Anxiety and fear deter women with intellectual disabilities from mammograms. - Inadequate care coordination and family support hinder screening. - Adult day programs lack mammogram education, contributing to knowledge gaps. - Physical inaccessibility and sedation issues pose challenges in mammogram accessibility. - Limited awareness about breast ultrasound’s role in screening.	****
(***: 60% quality, **** 80% quality, ***** 100% quality).					
